# Will the chemical probes please stand up?†

**DOI:** 10.1039/d1md00138h

**Published:** 2021-07-16

**Authors:** Ctibor Škuta, Christopher Southan, Petr Bartůněk

**Affiliations:** CZ-OPENSCREEN, National Infrastructure for Chemical Biology, Institute of Molecular Genetics of the Czech Academy of Sciences Vídeňská 1083 142 20 Prague 4 Czech Republic ctibor.skuta@img.cas.cz; Deanery of Biomedical Sciences, University of Edinburgh Edinburgh EH8 9XD UK

## Abstract

In 2005, the NIH Molecular Libraries Program (MLP) undertook the identification of tool compounds to expand biological insights, now termed small-molecule chemical probes. This inspired other organisations to initiate similar efforts from 2010 onwards. As a central focus of the Probes & Drugs portal (P&D), we have standardised, integrated and compared sets of declared probe compounds harvested from 12 different sources. This turned out to be challenging and revealed unexpected anomalies. Results in this work address key questions including; a) individual and total structure counts, b) overlaps between sources, c) comparisons with selected PubChem sources and d) investigating the probe coverage of druggable targets. In addition, we developed new high-level scoring schemes to filter collections down to probes of higher quality. This generated 548 high-quality chemical probes (HQCP) covering 447 distinct protein targets. This HQCP collection has been added to the P&D portal and will be regularly updated as established sources expand and new ones release data.

## Abstract 2

This study provides a comprehensive and comparative overview of probe sources, structures and targets. The analysis encompasses 4466 chemical probe candidates supported by evidence of specific binding to 796 human proteins.

## Graphical abstract

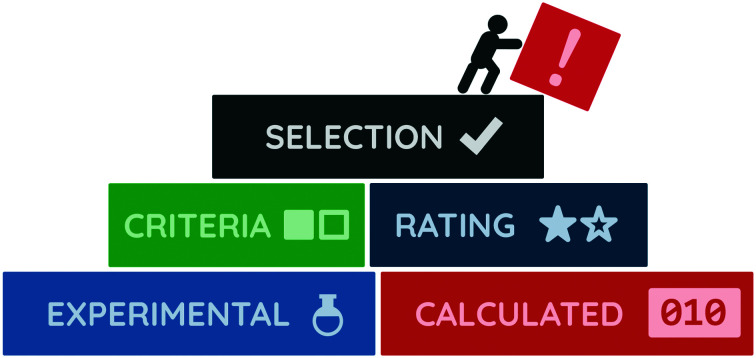

## Introduction

In 2005, the NIH Molecular Libraries Program (MLP) undertook the first large-scale identification of tool compounds to expand biological insights, now termed small-molecule chemical probes.^1,2^ Their systematic generation against a range of molecular targets was a key driver for the establishment of the PubChem database in order to collate structures and data from the initial ten funded screening centres.^3^ The concomitant screening compound collection was established as the Molecular Libraries Small Molecule Repository (MLSMR) for which PubChem had hosted 255 000 compounds by the end of 2005 and since expanded to 406 000 by 2015 (but updates have ceased). Although 25 of the 64 early compounds were judged to be of equivocal quality by a crowdsourcing assessment in 2009 (ref. 4) the program progressed to 375 probes (see data section below) before ending in 2014. As conceived from the outset, the availability of these compounds and, crucially, their associated characterisation data, have facilitated the exploration of new targets, pathways and therapeutic hypotheses.^5^ Notwithstanding these success stories, the MLP undertaking has been subject to criticisms that remain relevant to contemporary efforts. These include considerations of the MLSMR fitness-for-purpose as a library accrued in an academic context (compared to arguably better-resourced pharmaceutical company screening collections), persistent probe quality issues and remaining confusion on exactly how many probe compounds the program generated.^6–8^

A less tangible but equally important success of the MLP is that, from approximately 2010 onwards, it inspired other organisations to also initiate probe discovery with open dissemination. The three most recent announcements (but not yet surfacing data) are EU-OPENSCREEN^9^ with probe development as one of their main objectives, the EUbOPEN^10^ consortium aiming to synthesize at least 100 new chemical probes and the Target 2035 initiative to accrue probes for all human targets by 2035.^11^ As of June 2021, we were able to collect probe data from the sources listed below.

• MLP probes (NIH screening initiative)

• Structural Genomics Consortium (SGC, 3D structure-based)^12^

• Nathanael Gray Laboratory (cancer research focused)^13^

• Chemical Probes Portal (literature curation, expert opinion)^14^

• Pharmaceutical companies (offering in-house compounds)^15^

• Probe Miner (data filtration for putative probes)^16^

• Probes & Drugs (comprehensive collation of probe data)^17^

Detailed descriptions of these sources and their individual approaches to probe development are available from their websites and related publications. These are expanded in a recent review^18^ as well as articles in this special issue.

This work was conceived to answer the following questions that can not be answered *via* the individual sources:

• How many declared chemical probe structures are there?

• What is the distribution of their physicochemical properties?

• What are the differences between experimental and calculated probes?

• What is their representation in PubChem sources?

• What are their intersections with each other?

• What are their individual targets?

• What is their combined human proteome coverage?

Addressing these questions is specifically enabled by the Probes & Drugs portal (P&D, https://probes-drugs.org).^17^ P&D was designed as a hub for the integration of high-quality bioactive compound sets enabling their analysis and comparison. As the name indicates, the main focus is on probes and drugs but includes additional relevant sets extracted from, or supplied by, recently published specialist databases (*e.g.* BiasDB^19^), vendor sets, ESI† from papers (*e.g.* kinase inhibitors) or harvested from publication out-links (*e.g.* the British Journal of Pharmacology “Concise Guide” series^20–26^). Other high-quality curated bioactive chemistry sources, including ChEMBL,^27^ BindingDB,^28^ Guide To Pharmacology (GtoPdb),^29^ DrugCentral^30^ and DrugBank^31^ are utilized for compound biological annotation (the latter 3 are also P&D compound sets). The P&D compound database is currently composed of 69 sources including 12 probe-related, 7 drug compilations, 36 academic (non-commercial) sets and 14 precompiled sets from bioactive compound vendors (suggestions for expansion are welcome).

P&D has additional advantages for this study. An important one is that chemical structures from all sources are standardised after importation. This means that our internal comparisons are as rigorous as we can make them (notwithstanding cheminformatic nuances that preclude this from being perfect). We have used the *standardiser* python package^32^ for salt-striping, charge neutralization, standardizing common functional groups, preserving stereochemistry and identification of the main active pharmaceutical ingredients (API) in mixtures. Structural uniqueness is based on the InChIKey, a hashed version of the International Chemical Identifier, InChI.^33^ Within the text of this article, we have used InChIKeys to designate mentioned compounds. These can be searched not only against P&D and all major databases but also in Google.^34^ On our website, users can choose to browse and compare the structures in three different forms: 1) standardized; 2) original (*i.e.* as imported from the source) and 3) non-isomeric (*i.e.* core connectivity without stereochemistry). Importantly, we made the considered decision not to submit our entire content to PubChem for two main reasons. The first is to reduce non-obvious circularity which can confound database users.^35,36^ The second is that this enables informative Boolean query combinations to be made between P&D sets and essentially any PubChem source or filtration selects.

As of version 02.2021 (March 2021), P&D contains 77 130 compounds with 4466 labelled as chemical probes by their sources used for this study. However, these should not be considered equivalent in a canonical sense because they have been generated by divergent approaches. By analysing the structures and associated metadata, we have tried to establish a high-quality subset based on the probe origin and by the application of scoring schemes. In addition, where the data allow plausible assignments, we have compiled target coverage.

## Experimental *vs.* calculated

We chose to internally partition P&D probes into two main categories; experimental and calculated.

Experimental denotes compounds from published probe characterisation experiments. These papers include profiling data for target modulation potency, selectivity, and possible secondary targets. Also important to note is that the experimental data largely originate from a single laboratory and are thus likely to have more consistent and reproducible data (*e.g.* where intra-laboratory assay variation is controlled *via* sufficient replicates and internal standards). Examples of such experimental probe sets include; bromodomains chemical toolbox,^37^ Chemical Probes Portal,^14^ Gray Laboratory Probes,^13^ Nature Chemical Biology Probes, Open Science Probes,^15^ opnMe Portal,^38^ Protein methyltransferases chemical toolbox,^39^ and SGC Probes.^12^

We use the term calculated here to denote *in silico* evaluation using the combination of public data with a custom scoring function (we intentionally avoided the term predicted in this context because machine learning methods were not used).^16,40^ The evaluation of probes at scale involves comparing public data at different stringencies according to availability. The seven key criteria are: 1) <100 nM target potency *in vitro*, 2) <1 uM for cell-based assays with evidence of direct target engagement, 3) target selectivity >100-fold, 4) absence of structural alerts indicating chemical liabilities 5) identification of an inactive analogue as control with significantly lower potency or inactive against the primary target,^41^ 6) an orthogonal probe with a different chemotype against the same primary target, and 7) SAR data to increases confidence in specific target modulation. The simplest approach to assigning a compound as a probe is to use these criteria (or a subset thereof). However, this can be nuanced by weightings (*e.g.* for potency and selectivity) as well as more complex scoring (*e.g.* functions favouring compounds with a wider range of target profiling data). In contrast to experimental probes, the data for the evaluation of the calculated probes from different sources can be less consistent and may not be acquired with the objective of developing and validating a probe *per se*. The calculated sources included here are from *Probe Miner*^16^ and the *tool compound set*.^40^

## Source descriptions and counts

Those compared in this study are listed in Table 1 with brief descriptions below.

**Table 1:** Sources with their compound numbers and probe type. “E” refers to experimental and “C” to calculated probe type. The targets column counts distinct probe-target annotations. The total number of compounds/targets represents a distinct number of compounds/targets for all sets combined

	Set	Probe type	Set class	Compounds	Targets
1	Bromodomains toolbox	E	High-quality	25	26
2	Chemical Probes.org	E	High-quality	362	322
3	Gray Laboratory	E	High-quality	53	56
4	MLP	E	Legacy	375	156
5	Nature Chemical Biology	E	Legacy	58	51
6	Open Science Probes	E	High-quality	83	95
7	opnMe Portal	E	High-quality	55	57
8	Probe Miner	C	Calculated	3187	326
9	Methyltransferases toolbox	E	High-quality	19	20
10	SGC Probes	E	High-quality	81	97
11	Tool compound set	C	Calculated	515	392
12	Historical compounds	E	Historical/obsolete	239	—
	**Total**		**(Not historical)**	**4466**	**819**

### MLP and Nature Chemical Biology Probes

The former has been outlined in the introduction. The latter was extracted from articles published in Nature Chemical Biology (although since 2018 this dedicated section of the Journal is no longer available) As legacy collections, probes from these two sources may lack the stringent characterisation of maintained collections. This is reflected in some cases by a) the lack of controls or orthogonal probes, b) unclear potency and selectivity criteria or c) no target annotation.

### SGC Probes, Open Science Probes, opnMe Portal, and Gray Laboratory Probes

These organisations apply the currently accepted probe quality criteria and are maintained sets in that P&D has picked up at least some new compounds since their initial release (even if at a variable frequency).

### Bromodomains and protein methyltransferases chemical toolboxes

These compounds were extracted from publications focused on the study of bromodomains and methyltransferases. Except for four bromodomain probes developed elsewhere, these also belong to the *SGC set*.

### Tool compound set and Probe Miner

These are calculated selections, mainly from ChEMBL, selected *via* probe-likeness criteria. While the tool compound set is a one-off extraction from the publication, Probe Miner is regularly updated. The tool compound set independently includes more than 100 compounds available from the Chemical Probes Portal at the time of its publication.

### Chemical Probes Portal (CP portal)

This provides expert usage recommendations based on publication evaluations from a Scientific Advisory Board (SAB, of which one of us, CS, is a member). While content had languished below 200 compounds for some time, this has recently expanded to 362. Importantly, this portal also lists historical probes (these are also captured as a P&D set, see next section).

### Historical compounds

The use of these obsolete compounds is no longer recommended by the CP portal because new data indicates promiscuity or displacement by better tool compounds.^42^ These structures include the well-known and notorious medicinal chemistry time-wasters of staurosporine (HKSZLNNOFSGOKW-FYTWVXJKSA-N), quercetin (REFJWTPEDVJJIY-UHFFFAOYSA-N), resveratrol (LUKBXSAWLPMMSZ-OWOJBTEDSA-N), and curcumin (VFLDPWHFBUODDF-FCXRPNKRSA-N).

### Set comparisons

The sets are compared by exact matches in Table 2. The resulting matrix is unique in that no individual source has published a comparable analysis. However, some results were unexpected. The first surprise was the low intersection between MLP and the calculated sets. We attribute this to the 80 compounds from the MLP set without bioactivity data on P&D. In addition, 203 have a primary target potency above the 100 nM threshold. The unexpectedly high overlap between the Chemical Probes Portal and tool compound sets is a consequence of the (already mentioned) inclusion of the former in the latter but without data-supported evaluation. Also surprising is the low overlap between the two calculated sets, even though the data sources and selection criteria were conceptually similar. However, the main goal of the tool compound set was to select effective agonists or antagonists with a high stringency for target selection and cell potency.

**Table 2:** A matrix showing the intersections between 12 sources. This was computed using the InChIKey exact match for the standardised structures from the P&D portal. The diagonal figures in white represent the source counts in Table 1

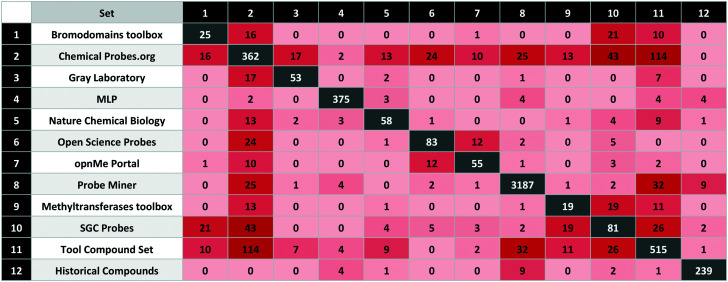

Another surprising observation was that, while overlap with historical compounds is reassuringly low, Table 2 indicates there are still nine of these undesirables in Probe Miner, four in the MLP, and two in the high-quality SGC Probes. The first of these, bromosporine^43^ (UYBRROMMFMPJAN-UHFFFAOYSA-N), was designed to be a pan-bromodomain inhibitor and could usefully be family-selective. The second, GSK-J1 (ref. 44) (AVZCPICCWKMZDT-UHFFFAOYSA-N), an inhibitor of the KDM protein family is not cell-permeable. The SGC Probes resource has noted this and consequently now recommends a pro-drug of GSK-J1, GSK-J4 (WBKCKEHGXNWYMO-UHFFFAOYSA-N) for cell-based assays.

## Dataset compilation

Merging individual sets resulted in 4466 structurally distinct probe compounds (*i.e.* as unique InChIKeys). This includes 940 (21%) experimental plus 3670 (82.2%) calculated probes including 3178 from Probe Miner. The overlap of 143 (3.2%) compounds is mainly due to the inclusion of the Chemical Probes Portal in the tool compound set. The full 4466 set includes 275 labelled as drugs (reported to be in clinical phases) with 103 labelled as approved by FDA, EMA and other agencies. The full set also includes 29 PROTACs (Proteolysis Targeting Chimeras) from PROTAC-DB^45^ and Chemical Probes Portal, 60 covalent binders from CovalentInDB,^46^ and 21 biased GPCR ligands from BiasDB.^19^

**Fig. 1:**
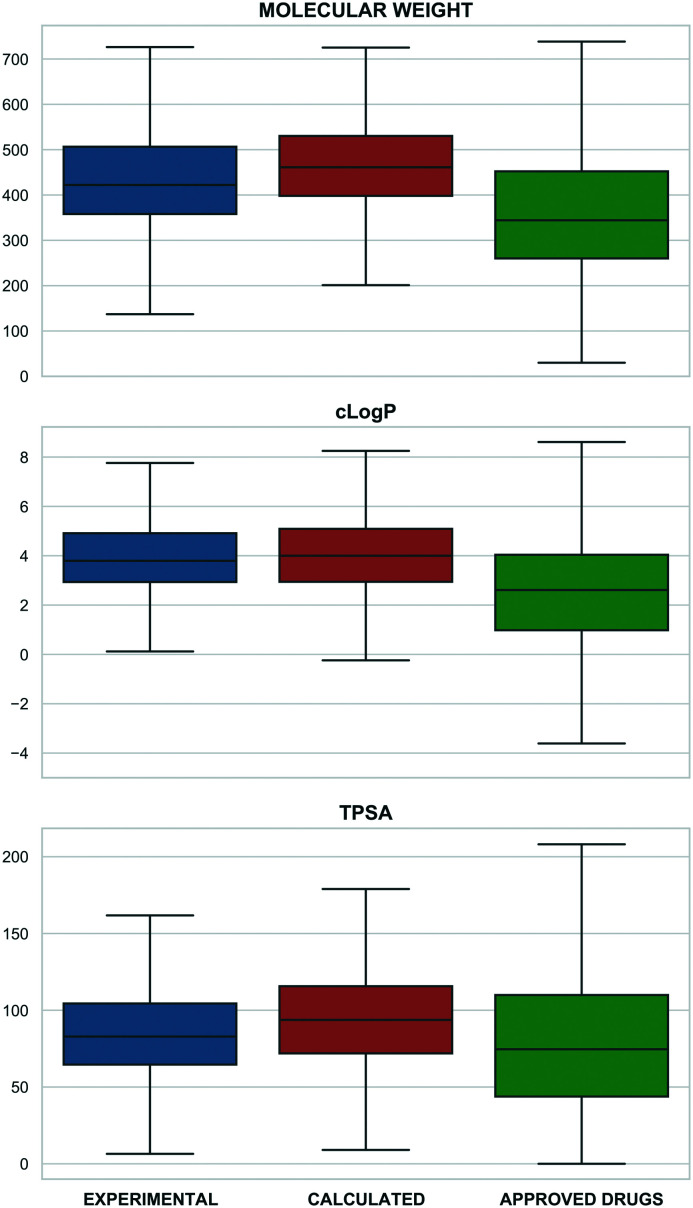
Physico-chemical properties distribution (top: molecular weight, middle: calculated log *P*, bottom: TPSA) for experimental probes (blue), calculated probes (orange) and small-molecule approved drugs set from ChEMBL (green) (*x*: property range, *y*: compounds percentage). The properties were calculated by RDKit.

Our analysis also established that 132 compounds were flagged with one or more structural alerts from either a) PAINS filters,^47,48^ b) aggregators,^49^ c) cellular assay nuisance compounds^50^ or d) historical compounds. Of the 60 stereoisomers, 54 originate from the Probe Miner set. Compared to small-molecule approved drugs extracted from ChEMBL (as a set in P&D) probes are generally larger and more complex (Fig. 1).

**Fig. 2:**
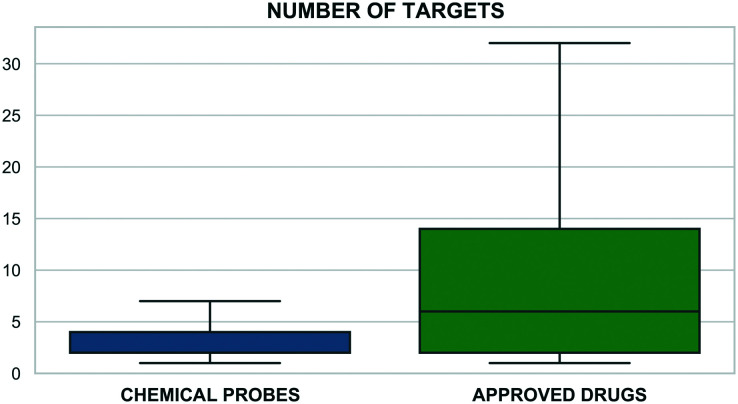
The number of associated targets for probes (blue) and the small-molecule approved drugs set from ChEMBL (green) (*x*: axis number of targets, *y*: compounds percentage). Only compounds with at least one associated target were included encompassing 4257 probes and 2005 approved drugs.

This correlates with higher target selectivity that, in turn, is reflected in the number of associated targets (Fig. 2). However, these values could be biased by approved drugs accumulating more cross-screening data and hence a wider range of secondary targets.

### Target mapping

The majority of probes have primary targets specified in their sources. In most cases these are supported by quantitative *in vitro* binding data (*e.g. K*_i_, IC_50_ or *K*_d_). Some may also have secondary targets with a data-supported potency below that against the primary target (we have avoided using the term “off-target” since there are few cases where secondary targets have been mechanistically assigned as a side-effect or toxicity liabilities). The 132 probes without primary target annotation were, in most cases, directed against viruses, bacteria, cell lines or pathways. They also predominantly belonged to the MLP and Nature Chemical Biology legacy sets. For the remainder, we collated 819 single and multi-component protein targets with 549 for both experimental and calculated with an overlap of 279. In total these constituted 807 distinct single protein identifiers (*i.e.* UniProt IDs^51^), 544 for experimental and 535 for calculated probes with 272 in-common. The human Swiss-Prot target count was 796.

In practice, the number of protein targets is below 819, since multi-component targets may be variably annotated against either a protein subunit, the complex target, or both. For example, probes directed against the BCR–ABL1 fusion protein may be annotated with the fusion protein (of which there are several length forms in TrEMBL but not Swiss-Prot) or ABL1 (P00519) or, in the case of ∼20 probes from the Probe Miner set, BCR (P11274). Other complicating examples are the cyclin-dependent kinases (CDKs), where the probes may be annotated by sources with one of the human CDKs, a CDK in complex with a specific cyclin, or both.

**Fig. 3:**
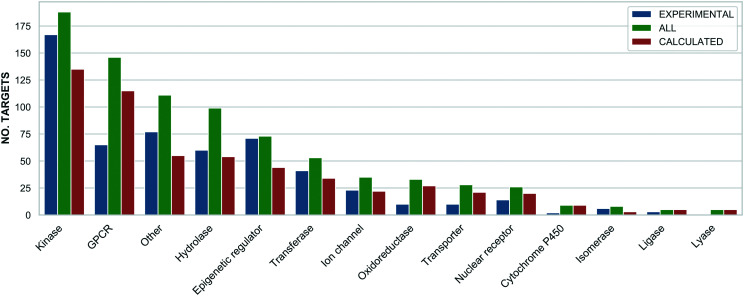
A bar chart showing the target families distribution separately for all (green), experimental (blue) and calculated (orange) probes (*x*: target family, *y*: number of targets). The assignments are based on the ChEMBL and Guide to Pharmacology target classification.

The highest probe target numbers (5 experimental and 225 calculated) have been assigned against mTOR (MTOR, P42345). Next in rank are histone deacetylase 1 (HDAC1, Q13547) with 2/161 experimental/calculated, epidermal growth factor receptor (EGFR, P00533) with 7/103 and estrogen receptor (ESR1, P03372) with 1/92. The larger number for calculated probes reflects their more frequent origin from panel screening papers and consequent higher average compounds: target ratio of 9.8 for the Probe Miner set compared to ∼1.0 for experimental probes. For these, the highest assigned target numbers are BRD4 (O60885) and the BRD3/BRD2 (Q15059/P25440) subfamily pair with 18 and 15 probes, respectively. This is a consequence of the declared SGC Probes focus on epigenetic regulators. The family distribution of all annotated targets is shown in Fig. 3.

The differences in Fig. 3 reflect inherent biases. For example, predicted probes have almost double the number of GPCRs^52^ but, compared to predicted probes, proportionally fewer kinases and epigenetic regulators. This is likely to be due to the challenges of optimising single-target selective ligands within these target families.^37,53^ However, probes with intra-family selectivity can also be experimentally useful but run the risk of being rejected by quality scoring weighted towards single-target selectivity.

### Target intersections in UniProt

**Fig. 4:**
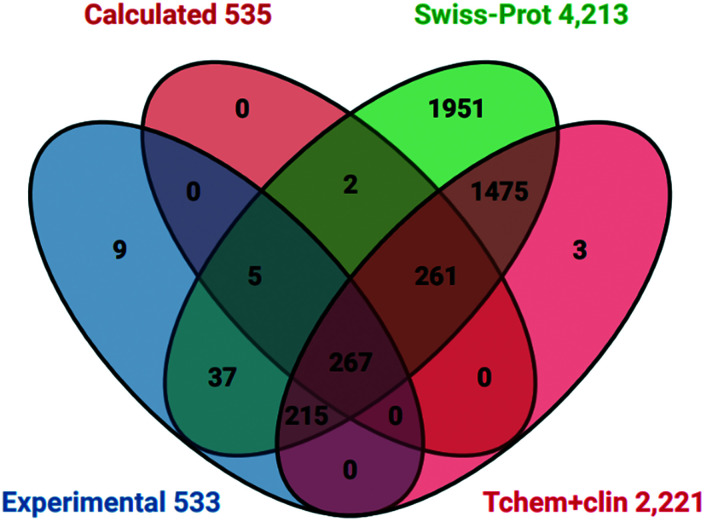
Venn diagram of P&D targets against selected human UniProt cross-references and two Pharos TDLs.

Having assigned target IDs to both probe sets we compared these with informative cross-references in UniProt.^51^ We selected 4213 protein IDs (as human Swiss-Prot entries) based on the union (OR operator) of the four high-quality curated chemistry-to-target databases (already mentioned) of ChEMBL, BindingDB, GtoPdb and DrugCentral. These IDs represent liganded targets for 21% of the UniProt proteome of 20 395 (although this drops to 19 205 for HUGO Gene Nomenclature Committee annotation). The comparative protein sets we also selected were from the four target development levels (TDLs) of the Pharos resource for Illuminating the Druggable Genome (IDG).^54^ This facilitates exploration of both the characterised and the understudied (or “dark”) regions of the human proteome with a view to expanding functional insights and finding new drug targets. We initially selected the combination of the Tchem^54^ (1593 proteins) known to bind small molecules (other than approved drugs) with target-class specific potency thresholds plus the Tclin^54^ (659 proteins) as targets of approved drugs. The union of these two is 2221 proteins. The intersections of these four lists are shown in Fig. 4.

The two notable features are:

1. Probes have activity against 53 proteins not in Tclin or Tchem.

2. The Swiss-Prot liganded proteome includes 743 probe targets.

Analysis with other TDL sets established that of the 659 approved targets 265 were also covered by probes. However, there were no intersections between probes and the 6368 Tdark proteins. This implies that the current probes may have already expanded Tchem but there is no overlap with dark targets.

### Source errors

Despite curatorial diligence, low levels of annotation errors, including the transitive inheritance of author mistakes extracted from papers, inevitably creep into the bioactivity databases we have used as sources.^55^ During manual cross-checking we identified the following error types affecting approximately 40 probe entries:

• Substitution of a biochemical for a cell-based assay as well as *vice versa* cases (the most common problem).

• Concentration unit errors for secondary target activity (*i.e.* the compound was thus not selective).

• Erroneous target annotations that falsely indicate potent secondary target activity.

• Potency values assigned to only a subunit in a multi-component target (*i.e.* thus technically without supporting bioactivity data).

• Some sources had incorrectly assigned human TrEMBL partial sequence entries as targets rather than the human Swiss-Prot IDs (although only three cases were found).

As an operating principle P&D fixes any unequivocal errors we spot. At the same time, we notify the originating sources about these errors that could otherwise persist and proliferate between databases. However, we have found the speed with which these are fixed at source has been variable (although this is clearly dependent on build cycle times and release versions).

## Probe scoring schemes

We optimised four different scoring schemes to support users for probe triage and selection. As explained, the Probe Miner (PMIS) and P&D probe-likeness scores (PDPS) are data-supported. The other two scores are expert opinion-based and thus more subjective. These are abstracted from the Chemical Probes Portal rating for use in cells (CPOC) and in organisms (CPOO). These represent a summed rating of chemical properties, primary targets, secondary targets and in most cases, expert judgments. The conceptual difference with data-supported scores is that these are calculated for compound-target pairs. Thus, a single compound can have multiple scores against each of its assigned targets. Users may thus select the most suitable of these pairings but the probes can also be used for intra- or inter-family selectivity. For comparability, all scores are normalized to between 0 and 100%.

The PMIS, ranging from 0 to 1, combines partial scores for 1) potency, 2) selectivity 3) activity in cells, 4) SAR data 5) availability of an inactive analogue and 6) a PAINS score. Nevertheless, probe suitability is not prescribed by the score value but by so-called minimum quality criteria. These include 100 nM potency in biochemical assays, 10-fold target selectivity, and 10 μM potency in cell-based assays (but not necessarily evidence of intra-cellular primary target engagement). The Probe Miner set of 3187 compounds meeting these criteria thus have a PMIS between 0.38 and 0.85. A more detailed description is given in the Probe Miner publication.^16^

The PDPS, scaled from 0 to 1, incorporates partial scores in common with PMIS but adds in orthogonal probes. The comparison of both scoring schemes is shown in Table 3. Unlike the Probe Miner selectivity score, P&D also highlights target sub-family selectivity beyond just single proteins. The probe-likeness of a compound is closely related to the PDPS value. Each compound with a score above 0.7 is labelled as P&D-approved based on the available data. The score is capped to not exceed 0.7 unless it passes all three core criteria (*i.e.*, *in vitro* potency, cell potency and selectivity). Compounds labelled as historical are down-weighted by subtracting 0.3 and thus cannot be labelled as P&D-approved. Currently, there are 1109 probes labelled as P&D approved. More details on this are given on the P&D FAQ page.^56^ The CPOC and CPOO scores (from 0 to 4 stars) are based on Scientific Advisory Board (SAB) reviews. These may be accompanied by comments and usage recommendations. However, there is currently a review backlog in that out of 362 compounds, 274 have been rated for use in cells and 225 for use in model organisms.

**Table 3:** Comparison of PMIS and PDPS. For parameters with a defined range, the score is 0% for values below the minimum and 100% for values greater than the maximum. Within this range, there is a linear relationship between the value and the score

Parameter	PMIS value range (**weight**) *note*	PDPS value range (**weight**) *note*
Potency (biochemical)	5–10 [−log(*M*)] (**4**)	6.5–7 [−log(*M*)] (**2**)
Selectivity	Complex selectivity score normalized per target (**8**)	10–30-Fold (**2**)
Potency (cell-based)	5 [−log(*M*)] (**2**) *without the evidence of primary target engagement*	5.5–6 [−log(*M*)] (**2**) *with the evidence of primary target engagement*
Inactive analogue	Binary (**1**)	Binary (**1**)
Orthogonal probe	—	Binary (**1**)
SAR	Binary (**1**)	—
Structural alert	Binary (**1**) *PAINS*	Binary (**1** and −**3** for historical compounds) *PAINS, aggregators + other nuisance compounds in cellular assays, historical compounds*
Probe-likeness determination	Independent of the score value, compounds labelled as possible suitable probes if they meet minimum quality criteria (100 nM potency, 10 μM cell potency, 10-fold selectivity)	Compounds labelled as P&D approved for PDPS >70%
Probe-like compounds count	**3187**	**1109**

In Table 4, we review three examples of compounds with assigned probe scores. The first is a selective RIPK1 inhibitor, *GSK2982772* (ref. 57) (LYPAFUINURXJSG-AWEZNQCLSA-N), highly scored by all three probe sources. The second is BET family bromodomain inhibitor from SGC Probes, *JQ-1* (ref. 58) (DNVXATUJJDPFDM-KRWDZBQOSA-N), scored highly by P&D and Chemical Probes Portal, but as a family-selective probe with lower PMIS. The latter is the only P&D approved probe with low ratings from the Chemical Probes Portal (not scored by Probe Miner), *AGI-5198* (ref. 59) (FNYGWXSATBUBER-UHFFFAOYSA-N), was proposed as a prototypical IDH-1 R132H inhibitor. However, this was surpassed in potency and characterization details by the more recent *GSK864* (ref. 60) (DUCNNEYLFOQFSW-PMERELPUSA-N) which also has an inactive control for proof of target involvement. However, as a second, distinct chemotype, this probe could be used for corroborative phenotypic assays.

**Table 4:** Three selected chemical probes with assigned probe scores from P&D, Probe Miner and Chemical Probes Portal

	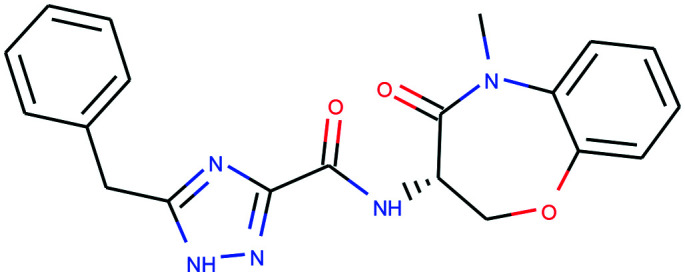	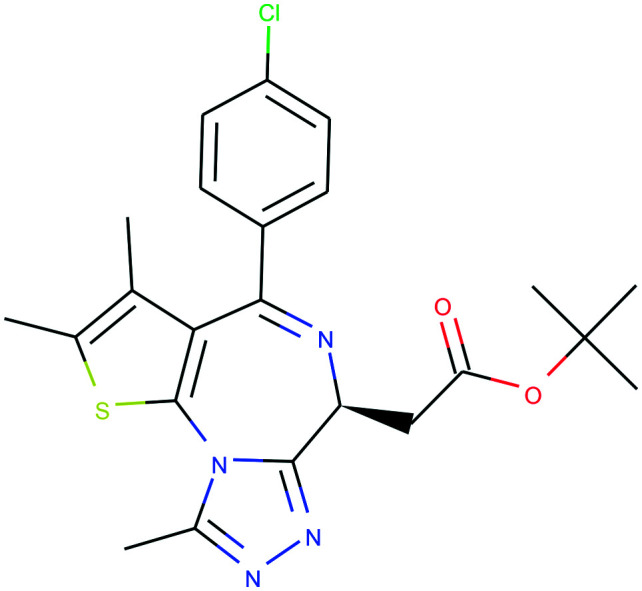	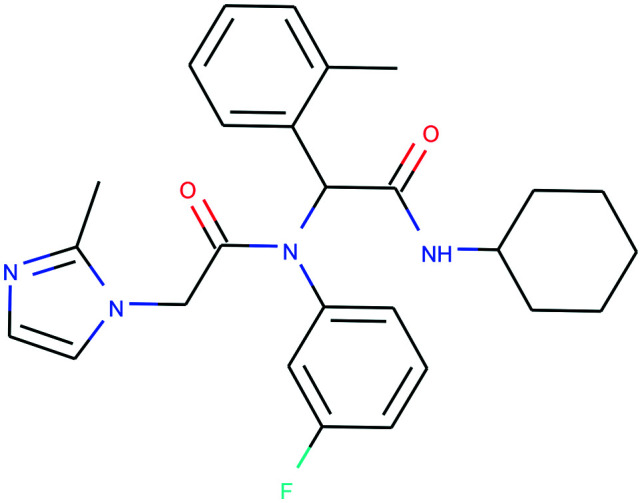
Name	GSK2982772	*JQ-1*	AGI-5198
PDPS	100%	100%	86%
PMIS	70% (*in Probe Miner set*)	48% (*not in Probe Miner set*)	—
CPOC	100%	100%	50%
CPOO	83%	75%	42%

### Score comparisons

To extend our systematic comparison of scoring, we introduced quality thresholds. PDPS was set at 70% as used for P&D approved probes. For CPOC and CPOO, we raised this to 75% (equivalent to 3 out of 4 stars in the original rating system). For the PMIS, there is no clear threshold and since the highest value is 85%, a setting of 75% would leave only 54 compounds from more than 3000. We thus chose to set the Probe Miner threshold at 60%, thus leaving 1282 compounds, a similar number to P&D approved probes.

The comparison between different scoring schemes (Table 5) highlights differences between judgment-based and calculated scores. One of the reasons for these differences is data incompleteness, for example where affinity data is only for the presumed primary target thereby precluding selectivity assessment.

**Table 5:** Matrix showing the intersections between six different probe scores and probe types. This was computed using the InChIKey exact match for the standardised structures from the P&D portal

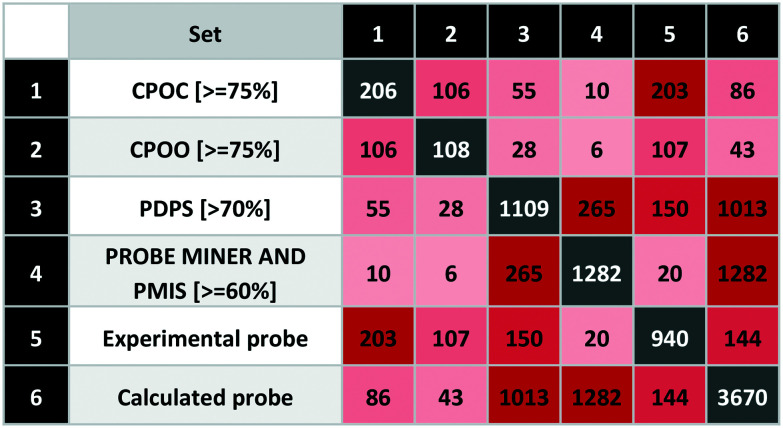

Even the experimental probes included 195 compounds without bioactivity data. In addition, we found 142 compounds annotated against single targets without selectivity data. The union of these represents 36% of the experimental probes that cannot thus be properly scored. Another reason for differences arises between stringent criteria-based evaluation and expert judgment.

We also found differences between calculated scores for the 265 compounds-in-common between the 1109 (P&D) and 1282 (Probe Miner) sets. This could be attributed to the differences in the scoring methodology but also the associated data (*i.e.* not all experimental probes have PMIS). While Probe Miner currently employs bioactivity data from ChEMBL and BindingDB, P&D uses more recent versions and complements these with smaller data sources such GtoPdb. This is reflected in a high P&D score for 150 experimental probes (with 55 highly-rated by CPOC) while Probe Miner detects 20 (including 10 based on the CPOC).

## High-quality chemical probes set

As an outcome of this study, we have compiled a high-quality chemical probes subset (HQCP). We have used the PDPS for the addition of P&D approved experimental probes plus those P&D approved calculated probes that are in at least one established tool compound set. We have thus partitioned four compound sets from P&D:

1. *Concise Guide to Pharmacology 2019/20* is a set extracted from a biennial series of publications providing concise overviews of the key properties of ∼1800 human drug targets with an emphasis on selective pharmacology.^20–26^

2. *Kinase chemogenomics set* is a collection of narrow-spectrum small molecule kinase inhibitors assembled by the SGC-UNC to study the biology of dark kinases. This is the most diverse and highly annotated public collection of kinase inhibitors.^61^

3. *Kinase inhibitors* were extracted from a series of Molecular Cell papers by Wang and Gray summarising recently-reported kinase inhibitors.^62,63^

4. *Novartis Chemogenomic Library – NIBR MoA Box* was compiled *via* data mining and institutional crowdsourcing. It is regularly updated and used widely both within Novartis and by their external collaborators.^64^

We used the quality criteria in Table 6 to select 548 probes for HQCP (451 experimental, 208 calculated with 114 in common). The intersections are shown in Table 7 including the EU-OPENSCREEN Bioactive Compound Library and Drug Repurposing Hub^65^ set. As non-commercial bioactive libraries, these are included in P&D as relevant for probe research.

**Table 6:** The criteria used for the selection of HQCP. The count column contains the number of compounds matched by the criterion. The total number represents the union of all criteria

	Criterion	Count
1	Belong to one of the high-quality probe sets (except Chemical Probes Portal)	**256**
2	CPOC or CPOO score at least 75% (*i.e.* three out of four stars in the original Chemical Probes Portal rating system)	**208**
3	P&D approved experimental probes	**150**
4	P&D approved probes belonging to one of the non-commercial high-quality sets (*Concise Guide To Pharmacology, Kinase Chemogenomic Set, Kinase Inhibitors, and Novartis Chemogenetic Library*)	**177**
5	Not labelled as a historical compound	**−2**
	**Total**	**548**

**Table 7:** Matrix showing the intersections between HQCP and other selected sets. This was computed using the InChIKey exact match for the standardised structures from the P&D portal

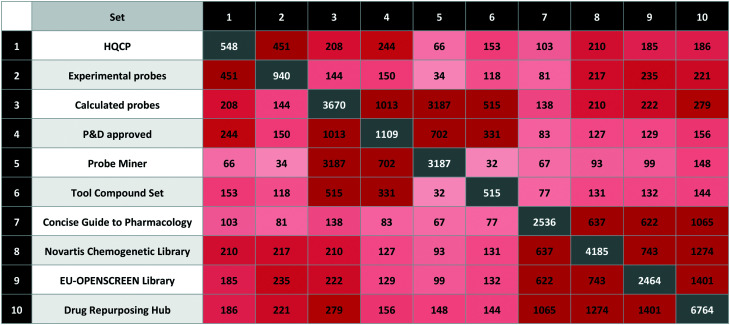

The HQCP set contains 42 approved drugs with 102 clinical candidates, 27 PROTACs, 15 covalent binders and 10 compounds tagged with a structural alert (four for aggregation and six for PAINS). The overlap between HQCP and the calculated sets is largest for the P&D approved probes with 244 out of 1109 compounds, but these were also partly used for the HQCP selection. From the complete Probe Miner set (*i.e.* without the PMIS threshold applied), there are 66 compounds from nearly 3200 meeting the Probe Miner minimum quality criteria. On the other hand, there are 153 compounds from 515 in the tool compound set, mainly from the inclusion of the Chemical Probes Portal compounds in the tool compound set.

The intersections between the three bioactive screening libraries (Novartis Chemogenetic Library, EU-OPENSCREEN Bioactive Compound Library, Drug Repurposing Hub) are 5.0%, 7.5% and 2.8%, respectively.

For target assessment, the HQCP covers 447 distinct proteins. The distribution of target families for all, experimental, calculated and HQCP probes is shown in Table 8. The HQCP was added as a separate compound set to the P&D portal and will be updated regularly. As new bioactivity data and new versions of compound sets are integrated we expect the number to increase.

**Table 8:** The target families distribution separately for all, experimental, calculated and HQCP probes

Target family	All	Experimental	Calculated	HQCP
Kinase	201	179	146	152
GPCR	146	65	115	70
Hydrolase	98	59	54	36
Epigenetic regulator	75	73	44	68
Transferase	39	28	23	26
Ion channel	34	22	21	16
Oxidoreductase	33	10	27	8
Transporter	29	11	22	12
Nuclear receptor	26	14	20	15
Cytochrome P450	9	2	9	3
Isomerase	8	6	3	2
Ligase	5	3	5	2
Lyase	5	0	5	0
Other	111	77	55	37
**Total**	**819**	**549**	**549**	**447**

## PubChem intersections

As the *de facto* global hub for chemical structures, associated bioactivity data and a massive range of informatic connectivity it was of considerable interest to profile probe sets against the 110 million compounds in PubChem.^66^ The first part of this necessitated the mapping of all P&D probe structures to PubChem CIDs *via* InChIKey matches and SMILES strings for cross-corroboration. This was done using the PubChem Identifier Exchange Service.^67^ We expected high coverage from the probe sources which we knew to have entered PubChem by various routes. From the 940 experimental probes, we recorded 915 CID matches (910 from IKs plus five more from SMILES). Inspection of the unmapped probes confirmed that most from SGC Probes, opnMe Portal and Gray Laboratory had no submission path into PubChem (directly, or *via* other source). In addition, we found the three unmatched MLP probes had different or flattened stereochemistry in PubChem (*i.e.* matched different non-isomeric CIDs). The corresponding 3670 calculated probes matched 3557 CIDs. While the mismatches were still only 3%, the reasons behind these are (again) differences in the handling of stereochemistry between PubChem and P&D (the latter uses RDKit as its main cheminformatics framework^68^). We also discovered that the links to some compounds are missing from ChEMBL because of InChIKey differences (ChEMBL is also using the RDKit framework^69^).

The second part of this analysis compared the two probe sets with selected PubChem sources to give additional insights. The numbers, shown in Table 9, are, again, a mixture of the expected and unexpected. We can propose explanations, starting with the experimental probes. The high level of BioAssay positive results is expected but does not establish whether those are the same probe-target pairs annotated in P&D.

**Table 9:** CID intersections between experimental and calculated probes for selected PubChem sources, ranked by the number of experimental probe matches. Note that most of these results can alternatively be read off directly from the P&D portal and give the same or close numbers. The total column represents a number of distinct compounds in the respective sources

Source	Total	Experimental	Calculated
PubChem	109 818 005	915	3557
BioAssays – active	1 457 929	800	3487
Vendors	59 867 622	784	810
ChEMBL	2 067 192	770	3519
Patents	39 401 959	652	2227
MLSMR	406 097	622	416
BindingDB	975 228	608	3331
GtoPdb	8705	305	335
PDBe	33 543	242	287
Chemical Probes Portal	467	186	131
BioAssays Probes	223	152	2

From the 915 CID matches in PubChem, 784 (85%) include vendors submissions, indicating a high availability for purchase. However, this expands slightly since additional vendor matches internal to P&D may not be indexed in PubChem (note also the opnMe probes are free upon application). The 770 matches in ChEMBL indicate high levels of probe-target activity data extracted from papers. However, there is an unexpected shortfall of 115 probes without any active results in BioAssay. The explanations are either the probe generators have not published in their assay results or these were not in journals that ChEMBL, BindingDB or the Guide to Pharmacology would have extracted and then submitted to PubChem.

The fact that 71% of the experimental probes have patent matches was a surprise since the impact of potential Intellectual Property (IP) issues on probe usage has not been widely discussed. While this high proportion seems at odds with the Open Science context that probe development teams espouse, the matches only mean the structures are specified in patent documents rather than necessarily being within the scope of allowed claims. Many of the automated extractions may merely represent prior-art mentions including where applicants have exploited analogue expansions from existing probe structures as drug discovery starting points. Notwithstanding, some probe structures may be explicitly claimed in maintained and granted patents (although precisely how many is difficult to assess). However, open patent information has become increasingly available and compound-to-patent document mappings are now indexed for nearly 40 million PubChem CIDs.^70^ An interesting example is the Boehringer opnMe GPR142 agonist *BI-1046* (MLOGCHDCTRINMU-UHFFFAOYSA-N). Two sources in PubChem have extracted the structure from Boehringer's WO2020007729 “Triazole benzamide derivatives as GPR142 agonists”. From CID 146293963 (*via* SureChEMBL SID 405725530), we can map the structure to example 2 and a table of low nM IC50 SAR values for 20 analogues (with synthesis details) that can also be found in the PubChem “Similar Compounds” section.

While the opnMe portal magnanimously declares that results generated with their molecules belong to the ordering scientists, the IP situation regarding other probe structure patent holders can only be addressed on a case-by-case basis. The assumption of Research Use Exemption should apply to US academics but the position of commercial institutions is less clear.^71^ Note, however, despite the detailed data package in the opnMe portal, the absence of a publication (BI-1046 is PubMed-negative but has over 40 false positives in Google Scholar because of an HIV clinical trial designation BI 1046) means that CID 146293963 has neither ChEMBL nor BioAssay links.

The 67% inclusion in the MLSMR means these particular probes may have expanded profiling data from unpublished assays not captured by ChEMBL, including testing against malaria, other disease parasites and cancer cell lines (this is particularly the case for the older MLP compounds). The extensive data overlap between ChEMBL and BindingDB arises from their mirroring collaboration but the latter has unique content from patent SAR extractions. For some years GtoPdb has included probe curation from papers selected for their pharmacological relevance and this is reflected in the capture of 305 probes.^29^ The availability of a PDB ligand structure for 242 probes is clearly enabling for many reasons but note these may not all be for the probe-primary target pair or species. The explanation for the low hits to the Chemical Probes Portal was the inclusion of historical probes in their 2017 PubChem submission (we suggest the separate submission of this cautionary subset in the future). The last row in the table presents two anomalies. As discussed above, at least 100 additional nominal MLP probes can be found in various lists beyond the 223 in the PubChem CID select for “BioAssay, Probes”.^7^ While reasons for the low match in P&D are being investigated the historical confusion associated with legacy MLP compounds may confound explanation.

The explanations for the calculated probes are the same as for the experimental but show a different pattern in the 11 rows of Table 9. Since these are predominantly derived from ChEMBL, the matches against this source, BioAssay active and BindingDB are all high. In contrast, vendor matches are proportionally much lower. While the patent intersection drops to 61% this still impacts 2227 CIDs. The explanation lies in the fact that many of the organisations (academic or commercial) generating the medicinal chemistry papers that ChEMBL curates (and Probe Miner selects) also file patents on their characterised compounds in advance of publication. Notwithstanding potential IP complications, it is important to note that patent matches are potentially advantageous for probe evaluation because they may well contain unpublished selectivity and SAR data not captured in probe sources.^70^

## Discussion

This work provides a uniquely comprehensive and comparative overview of probe sources and targets. This will be maintained and expanded for experts and non-informaticians seeking probes to use in their work. Although our results are presented in good faith, we understand the causes of fuzziness (some of which have been discussed) that caution against these numbers being taken as ground truth. Notwithstanding, we have analysed 940 experimental and 3670 calculated probe candidates. Together these provide evidence of specific binding for 796 human proteins across the target classes. We have flagged unsuitable (*i.e.* potentially misleading and resource-wasting) compounds from both probe groups. Compared to ChEMBL approved drugs, probes tend to be larger and more complex structures.

Although calculated probes are in a large majority, we established that their scoring is influenced by methodology and biases in data sources. Consequently, the application of PMIS and PDPS scoring retrieves different numbers of quality-rated probes from the Chemical Probes Portal set (*i.e.* 6 : 1 in favour of PDPS). We thus support scoring as a pragmatically useful means of compound prioritisation. By combining established criteria, we developed this further to delineate 548 high-quality chemical probes (HQCP) covering just under 450 targets. As we shown above, the Swiss-Prot bioactive chemistry cross-references indicate a data-supported druggable proteome of 20%. The current “probe proteome” targets would reach only 4% dropping to half of that for the HQCP set.

During the course of this work and the preceding years of P&D operation, the team has encountered a range of technical challenges most of which have been alluded to above. In this regard, while most stand-alone probe sources are designed with the needs of their users in mind, it is important for scientists to be able to navigate across multiple sources to obtain an overview of all potential probes in advance of experimental planning. This presents a particular challenge for non-informaticians and for which we needed much data-wrangling effort to complete the overview that P&D now offers.

During this work, we also detected problematic anomalies, some of which are listed below. These are not presented as criticisms but more as pointers towards what could be improved.

1. The current probe data landscape is particularly patchy. This means for many compounds their associated data falls short of the well-publicised criteria and thus compromises the utility of scoring.

2. Comprehensive characterisation of a probe, including the necessary broad cross-screening, requires extensive experimental work. In addition, the results need to be accessible (ideally in an open-access text-minable publication), reproducible and easily captured for transfer into database records. This situation can obviously be ameliorated by generating more data but, going forward, it is not clear how the existing data gaps can best be backfilled.

3. The bias towards known targets seems counter-intuitive. Given that mTOR has 38 020 PubMed hits and ChEMBL has 4557 compounds aligned against P42345 (including 20 clinical candidates), the need for 5 experimental and 225 calculated probes is not obvious (although new highly selective and potent allosteric modulators of old targets could provide new insights). As the Pharos TLD categories indicate, probe development that would broaden Tchem and make inroads into Tdark could lead to functional illumination (but with the caveat of the obvious paucity of assays for understudied proteins).

4. The identification and provision of the crucial control compounds lag behind probe availability. Notably, a recent analysis of negative controls extols the council of perfection in that a quartet of compounds is needed to maximise interpretation (*i.e.* two probes of different chemotypes and two negative controls, also matched as different chemotypes).^41^

5. There are many selective and potent compounds appearing in the recent medicinal chemistry and chemical biology literature that, while not officially yet declared in probe sources, include sufficient characterisation for useful probe criteria scoring. However, the rate of data extraction from these publications and flow into databases remains slow.^72^

6. We are considering how to address the mismatched and missing probes in PubChem but we need to iterate with the originating sources in the first instance.

## FAIR and reproducible

We have endeavoured to make this work findable, accessible, interoperable and reusable, according to Open Science principles.^73^ As for P&D itself, the licence is CC BY-SA 4.0. We have submitted a ESI† sheet that includes compound names, SMILES, InChIKeys, target identifiers, source assignments as well as other data used for the study. For interoperability, this will be deposited into Figshare in *.xlsx* format. No proprietary software has been used in this work and we thus expect any analysis reported here to be reproducible (if users encounter difficulties, they are welcome to contact us). As mentioned, all the intersections between sources inside the P&D database can simply be read off, combined, downloaded and users' own sets uploaded for further intersection analysis. Also as described, we recommend the PubChem Identifier Exchange Service for casting SMILES or InChIKeys at a medium scale against PubChem in total or selected sources within it. Many of the data sources used in this work (and consequently P&D) will expand with new releases so we expect numbers to change within a few months of these compilations made in April 2021.

## Author contributions

CŠ collated the data with CŠ and CS being responsible for the analysis. All authors edited and approved the final manuscript.

## Conflicts of interest

There are no conflicts to declare.

## Supplementary Material

MD-012-D1MD00138H-s001
